# On the limitations of some popular numerical models of flagellated microswimmers: importance of long-range forces and flagellum waveform

**DOI:** 10.1098/rsos.180745

**Published:** 2019-01-16

**Authors:** C. Rorai, M. Zaitsev, S. Karabasov

**Affiliations:** 1 School of Engineering and Materials Science, Queen Mary University of London, Mile End Road, London E1 4NS, UK; 2 Nuclear Safety Institute, ul. Bolshaja Tulskaja, 52, 115191 Moscow, Russia

**Keywords:** flagellated microswimmers, Stokes equations, sperm-cell swimming

## Abstract

For a sperm-cell-like flagellated swimmer in an unbounded domain, several numerical models of different fidelity are considered based on the Stokes flow approximation. The models include a regularized Stokeslet method and a three-dimensional finite-element method, which serve as the benchmark solutions for several approximate models considered. The latter include the resistive force theory versions of Lighthill, and Gray and Hancock, as well as a simplified approximation based on computing the hydrodynamic forces exerted on the head and the flagellum separately. It is shown how none of the simplified models is robust enough with regards to predicting the effect of the swimmer head shape change on the swimmer dynamics. For a range of swimmer motions considered, the resulting solutions for the swimmer force and velocities are analysed and the applicability of the Stokes model for the swimmers in question is probed.

## 1. Introduction

Flagellated microswimmers are cells or micrometre-sized robots that swim by moving appendages called flagella. Bacteria flagella appear as helical filaments rigidly rotated by a motor complex attached to the cell wall, eukaryotic flagella, instead, move by propagating sinusoidal waves in a whip-like fashion. The reason for this difference lies in the specific structure of eukaryotic cilia and flagella: the axoneme [1]. The ability to model flagellated microswimmers mathematically and numerically is relevant to a variety of applications in the fields of biology, medicine, medical diagnostics and engineering [2]. Beside improving our understanding of the physical phenomenon, accurate models can inform the design of effective microfluidic devices to sort microswimmers by motility [3–5] or suggest the design of efficient artificial microswimmers [6].

In this study, we are specifically concerned with the hydrodynamical modelling of sperm-cell swimmers. The dimensionless ratio between the inertia and viscous forces acting on these cells, namely, the Reynolds number, is of the order of *Re* = *UL*/ν ≈ 10^−2^, while the ratio between the characteristic viscous time scale and the time scale representing the rate of deformation of the swimmer body, i.e. the frequency Reynolds number, is of the order of *Re*_*ω*_ = *L*^2^*ω*/ν ≈ 10^−1^. Here *L* ≈ 50 µm and $U\approx 2\times 10^{-4}\;\text{m}\;{\text{s}}^{-1}$ are the characteristic length and velocity of the swimmer, *ω* ≈ 32 s^−1^ is the beating frequency of the flagellum and *ν* = 10^−6^ m^2^ s^−1^ is the kinematic viscosity of water. For *Re* and *Re*_*ω*_ ≪ 1, the flow field is typically computed by integrating the Stokes equations in a time-independent zero-Reynolds number framework (e.g. [7,8]). It can be noted, however, that neither of the two standard Reynolds number definitions include any scale associated with a change of the swimmer shape such as a characteristic wavelength of the flagellum motion that is not uniquely defined by the beating frequency. Thus, in the latter case, the applicability of the common criteria of ignoring the unsteady and inertial effects based on the two Reynolds numbers being of o(1) can be debated. It can be noted that classical studies [9] avoid this controversy by considering simplified flagellated swimmer models, which, for example, cannot capture the hydrodynamically important details of the flagellum waveform near the open ends, and assume that the wavelength of the swimmer’s motion is equivalent to its linear size. Although for a simple sperm cell swimming along a straight trajectory the wavelength is more or less equal to the length of the flagellum, in general, for more complex trajectories of the sperm cell that includes sharp turns or other organisms and artificial swimmers this is not the case.

The resistive force theory (RFT) [10], whose foundations were laid in [11], is a further simplified model applicable to the motion of slender bodies of which beating flagella is a good example. The RFT neglects the long-range hydrodynamical interactions and evaluates the viscous forces exerted on the immersed body as a function of the local velocities only. This model presents many advantages: it has a low computational cost when compared with the numerical integration of the Stokes equations allowing for proof of concept calculations [12,13], and it is simple enough to serve as a starting point for further analytical derivations [14,15], yet its accuracy is debated. Early works discussed the best choice for the model parameters, namely the normal and tangential hydrodynamical friction coefficients; different proposals were put forward by Lighthill [16] and Gray & Hancock [17]. Recent experimental tests showed that either choices badly capture the behaviour of helical flagella for the range of shapes present in nature [18,19]. Other studies calibrated the parameters to match experimental observations [20] or the results obtained by integrating Stokes [21]. The necessity of calibrating the model versus experimental observations or the results of more sophisticated models highlights the unsuitability of the RFT approximation for applications outside the range of calibration.

The RFT is applied to the flagellum, the contribution of the approximately ellipsoidal cell-body or ‘head’ is evaluated through analytical expressions [22] and added to the flagellum contribution to compute the entire cell dynamics. On this same line, one may represent the flagellum by a model of choice and still approach the problem by separately studying the cell body and flagellum dynamics before simply summing their contributions [23]. This procedure is naturally embodied in the RFT and is, for example, implied by studies that look for the optimal flagellum shape, neglecting to include the head in the calculations (e.g. [10,14]).

In this paper, we study the motion of a single sperm cell in an infinite domain to address three issues: (i) the accuracy of approximating swimming as the linear superposition of the dynamics of separate body-parts (head plus flagellum) versus a full-body description, (ii) the accuracy of modelling swimming with the RFT, which is one of the possible approximations based on the above superposition that ignores long-range hydrodynamical interactions, and (iii) the validity of the quasi-steady and inertia-less assumption for swimming at the micro-scale, where the quasi-steady hypothesis entails assuming that the flow instantaneously adapts to the body deformations.

To address the first two issues, we contrast the results obtained by applying the simplified approaches and the full hydrodynamical model, §§3.1 and 3.2. In particular, we compare the swimming velocities, trajectory and force distribution on the swimmer body. We then study locomotion of swimmers with different head shapes, §3.3, and show that the simplified approaches fail to identify the most hydrodynamically efficient swimmer. To tackle the third point, we calculate the propulsive matrix, that is the matrix that relates the forces generated by the moving flagellum to the rigid-body velocities of the swimmers, and we analyse its eigenvalues and eigenvectors to identify a criterion that establishes when the inertia-less quasi-steady hypothesis is valid, §3.5.

It should be pointed out that investigation of the applicability limits of classical semi-analytical micro-swimmer models has been a popular topic since the original seminal papers by Hancock [11,17], who developed slender body theory (SBT) and its algebraic approximation known as the RFT. For example, Higdon [24] developed a version of the finite amplitude SBT to study the importance of taking into account head–flagellum interaction by considering an analytical Green’s function solution of the spherical head. The latter was superimposed on the flagellum solution without taking the effect of flagellum on Green’s function of the head into account, thus, ignoring some of the non-local hydrodynamic interactions. In the same work, the solutions of RFT with Lighthill’s coefficients were compared with the SBT results and large errors (25–50%) were reported in the case of sufficiently large-cell bodies relative to the flagellum length.

Johnson [25] developed a further advanced version of SBT through matched asymptotic expansions with the error term being quadratic in slenderness. This theory was used in the study of Johnson & Brokaw [9] to show that RFT is satisfactory for use in analysis of mechanisms for the control of flagellar bending. Along a similar line of research, Brokaw [26] simulated the behaviour of a spermatozoa flagellum by an active shear system controlled by the curvature of the flagellum. In that work, a good agreement of RFT and SBT was reported.

In accordance with the derivation assumptions [11], SBT models not only require a small thickness of the flagellum relative to its length, but also the amplitude of the displacement being small compared with the wavelength. Within the applicability range, however, SBT models showed excellent agreement with more sophisticated models such as regularized Stokeslet method (RSM) [18], boundary element method [27], finite-element method (FEM) [28], finite volume method solutions and the experimental data [29]. Notably, the test cases considered in these studies typically involve idealized models such as a small rigid helix spiral moving in water at a distance of at least one slender-body length from boundaries, which falls under remit of the SBT approximations. Later studies that focused on the dynamics of such swimmers close to no-slip and/or free-slip boundaries generally adopted a boundary element method approach [30–32].

Despite this extensive prior research on the zero-Reynolds number propulsion of flagellated microorganisms, a systematic comparison of RFT, which can be seen as an abridged version of SBT, and a direct solution of the Stokes equation in the case of a realistic, flexible flagellum of a sperm cell [12] with and without fully accounting for non-local hydrodynamic effects of the head/flagellum interaction, has not been performed yet. Neither has a systematic study of the waveform shape on the sperm swimmer’s dynamics been conducted with an examination of the common assumptions such as neglecting the flow unsteadiness in accordance with the Stokes’ flow assumption. Both of these are a distinct focus and novelty of the present paper.

The paper is organized as follows: in §2, we introduce the numerical methods used to simulate the flagellated swimmer motion. The RSM is presented in §2.1, the swimming problem details in the context of the RSM are discussed in §2.2, while in §2.3 the geometry and beating movement of the flagellum are defined. The application of a finite-element method for the same microswimmer problem is introduced in §2.4. In §3, we report the numerical results including a validation of the RSM versus the FEM code, a modal analysis of the swimming velocities and a visualization of the flow field induced by the swimmer (§3.4). We summarize the main results in §4.

## 2. Mathematical models

### 2.1. Regularized Stokeslet method

The fundamental solution of the incompressible forced Stokes equation

2.1 $$-\nabla p+\mu \nabla^{2}u+f\delta (x-y)=0$$

and

2.2 ∇⋅u=0,

for a point force **f** acting on **y** in an unbounded domain is the *Stokeslet* J(r)

2.3 $$u(x)=f\cdot J(r)=\frac{f}{8\pi \mu }\cdot \left[\frac{I}{r}+\frac{rr^{T}}{r^{3}}\right],$$

where *r* = |**x** − **y**|, **r** = (**x** − **y**), I is the identity matrix, *μ* is the dynamic viscosity of the fluid and *δ* is the *δ*-function.

Since the Stokes equation is linear, the flow field generated by an immersed body with a deforming boundary S(t) can be represented through a continuous distribution of Stokeslets [33]

2.4 $$u(x,t)=\int_{S(t)}f(y)\cdot J(r)\;\text{d}S_{y}.$$

For complex geometries, as is the case of flagellated microswimmers, the integral is computed numerically by discretizing the immersed surface: for *n*, the points on which the velocity is evaluated, *m*, the grid points on the surface of the immersed body and *A*_*m*_ the quadrature weights

2.5 $$u_{n}=\frac{1}{8\pi \mu }\sum_{m}\left(\frac{I}{\left|r_{nm}\right|}+\frac{r_{nm}r_{nm}^{T}}{|r_{nm}{|}^{3}}\right)\cdot f_{m}A_{m},$$

where **r**_*nm*_ = **x**_*n*_ − **x**_*m*_ is the radius vector from point *n* to *m*. In our calculations, the RSM grid is built on the surface of the swimmer head and on the cylindrical surface of the flagellum of radius *F*_*r*_, see §2.3 for details on the swimmer geometry.

The Stokeslets are singular kernels, their singularity can be dealt with by replacing the point force with an approximate point force with local support. In practice, as proposed by Cortez *et al.* [34], **f***δ*(**r**) can be replaced by **f***ϕ*_*ε*_(**r**)

2.6 $$\phi_{\epsilon }(r)=\frac{15\epsilon^{4}}{8\pi {\left(r^{2}+\epsilon \right)}^{7/2}},$$

yielding the *regularized* Stokeslet

2.7 $$J_{ij}^{\epsilon }=\delta_{ij}\frac{r^{2}+2\epsilon^{2}}{{\left(r^{2}+\epsilon^{2}\right)}^{3/2}}+\frac{\left(x_{n,i}-x_{m,i})(x_{n,j}-x_{m,j}\right)}{{\left(r^{2}+\epsilon^{2}\right)}^{3/2}},$$

for which 97% of the force is within a radius *ε* [34], where *ε* is the regularization parameter that requires calibration. By running some tests for the flow past an ellipsoid (see §2.2) we have found, consistently with [18], that our numerical results minimize the error when *ε* is between one-third and one-half of the grid spacing.

### 2.2. The swimming problem

The velocity of a swimmer in Stokes flow can be decomposed into a rigid-body translation *v*_*j*_, a rigid rotation *ω*_*j*_ and the body deformation (head and flagellum) $u_{\;j}^{BC}(x)$. For convenience, we express these velocities in the frame of reference of the swimmer. The head is non-motile ($u_{\;j}^{BC}=0$), while the flagellum moves with the beating motion introduced in §2.3. We consider the case of a flagellum beating on the *z* = 0 plane.

In the context of the RSM, the swimming problem is solved by inverting the system

2.8 $$u_{\;j}^{BC}(x)=\frac{1}{8\pi \mu }\sum_{n=1}^{N}\sum_{i=1}^{3}J_{ij}^{\epsilon }(x,x_{n})f_{n,i}A_{n}-v_{\;j}-\omega \times x\cdot e_{\;j},$$

with constraints

2.9 $$f_{n,i}A_{n}=0$$

and

2.10 $$F_{n,i}x_{n,j}\varepsilon_{ijk}=0,$$

to find the forces *F*_*n*,*i*_ and the swimming velocities *v*_*j*_ and *ω*_*j*_. Here *F*_*n*,*i*_ = *f*_*n*,*i*_*A*_*n*_, for *n* = 1, 2 … *N* and in equations (2.9) and (2.10) the summation over the repeated index convention is adopted, with *i* = 1, 2, 3 as well as *j* and *k*. The conditions (2.9) and (2.10) derive from the fact that forces and torques need to balance exactly since inertia is absent.

An alternative but equivalent approach consists in computing the propulsive matrix coefficients *C*_*x*_, *C*_*y*_, *m*_*z*_

2.11 $$\left[\begin{array}{ccc} C_{x}^{v_{x}} & C_{x}^{v_{y}} & C_{x}^{\omega }\\ C_{y}^{v_{x}} & C_{y}^{v_{y}} & C_{y}^{\omega }\\ m_{z}^{v_{x}} & m_{z}^{v_{y}} & m_{z}^{\omega } \end{array}\right]$$

and solving the system

2.12 $$C_{x}^{v_{x}}v_{x}+C_{x}^{v_{y}}v_{y}+C_{x}^{\omega }\omega =-F_{x}^{B},$$

2.13 $$C_{y}^{v_{x}}v_{x}+C_{y}^{v_{y}}v_{y}+C_{y}^{\omega }\omega =-F_{y}^{B}$$

2.14 $$\;\text{and}\;m_{z}^{v_{x}}v_{x}+m_{z}^{v_{y}}v_{y}+m_{z}^{\omega }\omega =-T_{z}^{B},$$

for *v*_*x*_, *v*_*y*_ and *ω*. The coefficients *C* and *m* are computed by solving the Stokes equation separately for an arbitrary (unitary for convenience) rigid translation of the swimmer body and an arbitrary solid body rotation. They correspond to the surface integral on the swimmer body of the *x*, equation (2.12), and *y*, equation (2.13), component of the force density *f*_*i*_, and the *z*, equation (2.14), component of the torque density for, respectively, a unitary *v*_*x*_, *v*_*y*_ and *ω*. The known terms $F_{x}^{B}$, $F_{y}^{B}$, $T_{z}^{B}$ are the integrals of the forces and torque due to the flagellum beating only. This approach requires solving equation (2.8) four times (for an arbitrary *v*_*x*_, *v*_*y*_, *ω* and for the flagellum beating) but has the advantage of producing better conditioned matrices. We recall that a direct consequence of the reciprocal theorem is that the resistance matrix (2.11) is symmetric.

If we approximate the swimming problem by treating the flagellum and the head separately, the propulsive matrix for the frame of reference located on the head centroid becomes

2.15 $$\left[\begin{array}{ccc} \left(C_{x}^{v_{x},t}+C_{x}^{v_{x},h}\right) & C_{x}^{v_{y}} & C_{x}^{\omega ,t}\\ C_{y}^{v_{x}} & \left(C_{y}^{v_{y},t}+C_{y}^{v_{y},h}\right) & C_{y}^{\omega ,t}\\ m_{z}^{v_{x},t} & m_{z}^{v_{y},t} & \left(m_{z}^{\omega ,t}+m_{z}^{\omega ,h}\right) \end{array}\right],$$

where the superscript ‘t’ and ‘h’ stand for the tail (flagellum) contribution and the head contribution. We will next refer to this approach as the head + tail (H + T) model.

If the head is spherical $C_{x}^{v_{x},h}=C_{y}^{v_{y},h}=6\pi \mu L_{\mathrm{head}}/2$ and $m_{z}^{\omega ,h}=8\pi \mu {\left(L_{\mathrm{head}}/2\right)}^{3}$. Expressions for $C_{x}^{v_{x}}$ $C_{y}^{v_{y}}$ and $m_{z}^{\omega }$ for a prolate ellipsoid with *b* = *c* as the minor semi-axes and *a* as the major semi-axis are derived in [22],

2.16 $$C_{x}^{v_{x}}=6\pi \mu a\frac{8}{3}e^{3}{\left[-2e+(1+e^{2})\log\frac{1+e}{1-e}\right]}^{-1},$$

2.17 $$C_{y}^{v_{y}}=6\pi \mu a\frac{16}{3}e^{3}{\left[2e+(3e^{2}-1)\log\frac{1+e}{1-e}\right]}^{-1}$$

2.18 $$\;\text{and}\;m_{z}^{\omega }=8\pi \mu ab^{2}\frac{4}{3}e^{3}\left(\frac{2-e^{2}}{1-e^{2}}\right){\left[-2e+(e^{2}+1)\log\frac{1+e}{1-e}\right]}^{-1},$$

where $e=\sqrt{1-{\left(b/a\right)}^{2}}$ is the eccentricity, and $m_{z}^{\omega }$ is calculated for a rotation about a minor axis.

**Figure 1.**
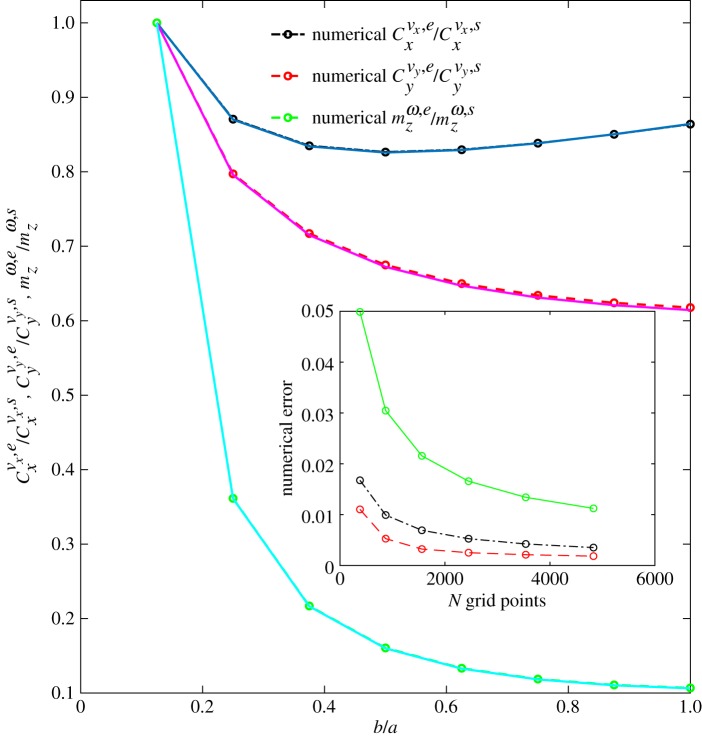
Ratio between $C_{x}^{v_{x}}$ for a prolate ellipsoid, namely $C_{x}^{v_{x^{,e}}}$, and $C_{x}^{v_{x}}$ for a sphere of equal volume, namely $C_{x}^{v_{x^{,s}}}$ (black dashed-dotted line with circles and solid blue line), similarly, the ratio $C_{y}^{v_{y^{,e}}}/C_{y}^{v_{y^{,s}}}$ (red dashed line with circles and magenta solid line) and $m_{z}^{\omega ,e}/m_{z}^{\omega ,s}$ (green dashed line with circles and cyan solid line). The curves are rescaled by their maximum value to facilitate a direct comparison between them. The solid lines correspond to the analytical solution of equations (2.16)–(2.18), while the dashed lines with empty circles represent the numerical solution for an ellipsoid discretized by 4694 points. The inset shows the numerical error for these same quantities as specified by equation (2.19) and as a function of the number of points the surface is discretized into.

To test the accuracy of the RSM, we study the flow past a prolate ellipsoid for *N*_*e*_ different stretching ratios, *b*/*a*, in the range 0.125 < *b*/*a* < 1. A comparison between the analytical result, i.e. expressions (2.16)–(2.18), and the numerical result obtained with the RSM is shown in figure 1. After calibration, the regularization parameter *ε* is chosen to be $\epsilon =0.5\sqrt{S/N}=0.5\sqrt{A}$, where S is the surface of the ellipsoid and *N* is the number of grid points the ellipsoid is represented by. The numerical error, plotted in the inset of figure 1, is computed as

2.19 $$\text{Error}=\frac{1}{N_{e}}\sum_{i}\frac{\left|C_{\mathrm{numerical}}^{i}-C_{\mathrm{analytical}}^{i}\right|}{C_{\mathrm{analytical}}^{i}},$$

where *C* stands for the coefficient $C_{x}^{v_{x}}$, $C_{y}^{v_{y}}$ or $m_{z}^{\omega }$ for a prolate ellipsoid divided by the same coefficient for a sphere of equal volume. Note that for a given volume, the surface area increases as the aspect ratio decreases.

We have compared the results for two different distributions of the grid points: the case of equally spaced points on the surface of the ellipsoid and an the case of an uneven grid (a discretization based on spherical coordinates with points located at equal azimuthal and polar angle intervals). We finally chose to adopt the latter since the results are not very sensitive to the type of grid once the resolution is large enough.

### 2.3. Swimming parameters

**Figure 2.**
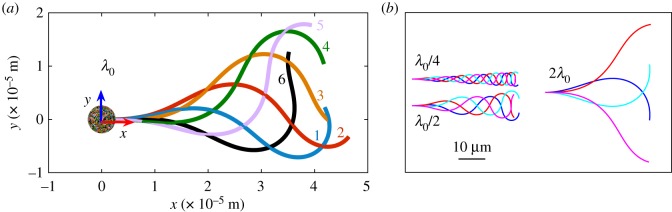
(*a*) Swimmer body shape at six evenly spaced times within one period of *π*10^−2^ s. The head is spherical of diameter *L*_head_ = 5 × 10^−6^ m, the flagellum length is 50 × 10^−6^ m, and the tail beating is given by formula (2.21). This shape corresponds to the reference wavelength λ_0_. (*b*) Flagellum centreline for four evenly spaced times within one beating period and three different wavelengths: λ = λ_0_/4, λ_0_/2, 2λ_0_.

We denote the mean flagellar curvature as *K*_0_, the flagellum frequency as *ν*, λ the wavelength and *A*_0_ the amplitude of the wave [20]. Following [21], we first use the values: *K*_0_ = 7735.5 rad m^−1^, *ν* = 200 rad s^−1^, λ = λ_0_ = 52.19 µm, *A*_0_ = 16828.83 rad m^−1^, which give a similar flagellum waveshape compared with [12]. Additionally, we consider the flagellum radius to be *F*_r_ = 0.25 µm, and the spherical swimmer head to have diameter of *L*_head_ = 5 µm (figure 2). We then study the case *K*_0_ = 0 which corresponds to a swimmer following a rectilinear rather than circular trajectory. Furthermore, to investigate the effect of the characteristic wavelength on the swimmer dynamics we perform some simulations with different λ: λ_0_/4, λ_0_/2, 2λ_0_, λ → ∞ (figure 2*b*).

Following [12,20], for an angle *Ψ* measured along the flagellum arc length *s* equal to

2.20 $$\Psi (s,t)=K_{0}s+2A_{0}s\cos\left(\nu t-\frac{2\pi s}{\lambda }\right),$$

the flagellum coordinates are

2.21 $$r(s,t)=\frac{L_{\mathrm{head}}}{2}e(t)+\int_{0}^{s}\cos[\Psi (u,t)]e(t)+\sin[\Psi (u,t)]e^{⊥}(t)\;\text{d}u,$$

where **e** = [cos*θ*, sin*θ*] and **e**^⊥^ = [ − sin*θ*, cos*θ*] with *θ* the angle between the chosen reference frame and the swimmer reference frame, the latter is shown in figure 2. In the frame of reference of the swimmer, base vectors are time independent and equal to **e** = [1, 0] and **e**^⊥^ = [0, 1], hence, the velocities of the flagellum are

2.22 $$u_{x}^{BC}(s,t)=-\int_{0}^{s}\sin[\Psi (u,t)]\dot{\Psi }\;\text{d}u,\;u_{y}^{BC}(s,t)=\int_{0}^{s}\cos[\Psi (u,t)]\dot{\Psi }\;\text{d}u,$$

with

$$\dot{\Psi }=-2A_{0}\nu s\sin\left(\nu t-\frac{2\pi s}{\lambda }\right).$$

These values, together with the null-velocity distribution on the swimmer’s head, form the known term of the system of equations (2.8).

As a remark, we stress that the swimming problem is typically solved in the frame of reference of the swimmer (e.g. in [12]) with the origin of the axes on the centre of the head as shown in figure 2. Although arbitrary, this choice is convenient since this is the natural frame to express the velocities of the flagellum. Note, however, that the centre of mass of the head is not the centre of mass of the entire body, the latter moves as the flagellum itself changes shapes and mostly falls outside the swimmer body. A different choice of the frame of reference leads to different values of forces, torques and velocities, but once the results are recasted in a common frame, e.g. the laboratory frame, velocities and trajectories coincide. This is a consequence of the fact that the torque for systems with null-force resultants is independent of the location of the frame of reference. In this case, the torque resultant is zero too.

When λ → ∞ the expressions for the coordinates simplify and the integrals in d*u* can be easily computed

$$r(s,t)=\left[\frac{L_{\mathrm{head}}}{2}+\frac{\sin(s\kappa )}{\kappa }\right]e+\left[\frac{1}{\kappa }-\frac{\cos(s\kappa )}{\kappa }\right]e^{⊥},$$

where *κ* = *K*_0_ + 2*A*_0_cos(*νt*). The boundary conditions result in the complex time-dependent functions

2.23 $$\begin{array}{rl} u_{x}^{BC}(s,t) & =2A_{0}\nu \sin(\nu t)\left[\frac{\sin(s\kappa )}{\kappa^{2}}-\frac{s\cos(s\kappa )}{\kappa }\right]\\ \text{and}\;u_{y}^{BC}(s,t) & =2A_{0}\nu \sin(\nu t)\left[\frac{1}{\kappa^{2}}-\frac{\cos(s\kappa )}{\kappa^{2}}-\frac{s\sin(s\kappa )}{\kappa }\right], \end{array}\}$$

where cos(*sκ*) and sin(*sκ*) can be expanded in a Taylor series to yield series of, respectively, even or odd powers of *sκ*. In the special case of the rectilinear swimmer, i.e. *K*_0_ = 0, the expansion simplifies into even, for $u_{x}^{BC}$, and odd, for $u_{y}^{BC}$, power series of 2*A*_0_*s* cos(*νt*) that correspond, in the frequency domain, to spectra with only even or odd modes different from zero.

After drawing the flagellum centreline, we use the local Frenet–Serret frame to build the cylindrical surface of radius *F*_r_, which we discretize by approximately evenly spaced points. The distance between the points is chosen in such a way that the corresponding surface area approximately equals the surface area A relative to the points on the head (see end of §2.2). In conclusion, the regularization parameter for the entire swimmer surface is $\epsilon =0.5\sqrt{A}$.

### 2.4. Finite-element method

To cross-verify solutions obtained with the RSM, the system of the governing three-dimensional Stokes equations

2.24 $$-\nabla p+\mu \nabla^{2}u=0$$

and

**Figure 3.**
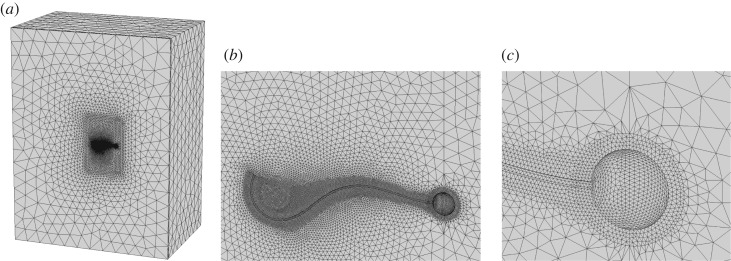
(*a*) Computational domain for the finite-element solution. (*b*) Zoomed finite-element mesh around the swimmer. (*c*) Zoomed finite-element mesh around the swimmer head.

2.25 ∇⋅u=0

is solved numerically in the reference frame fixed with the centre of the spherical head of the swimmer. The time period of the flagellum motion is discretized into 100 uniform time steps, which amount was found sufficient for accuracy. Each time moment corresponds to a particular configuration of the flagellum wave shape (2.20) and (2.21). For each shape of the swimmer, the same open-boundary computational box domain around the swimmer is specified. The box size is large enough to simplify the specification of numerical boundary conditions at the external boundaries. The domain is discretized by tetrahedral grid elements with applying a sufficient refinement near the swimmer boundary to resolve both the head and the flagellum surface. The total number of grid cells in the model is about 440 000 and the grid details are shown in figure 3. The grid is generated by using the software magentablack‘gmsh’.

**Figure 4.**
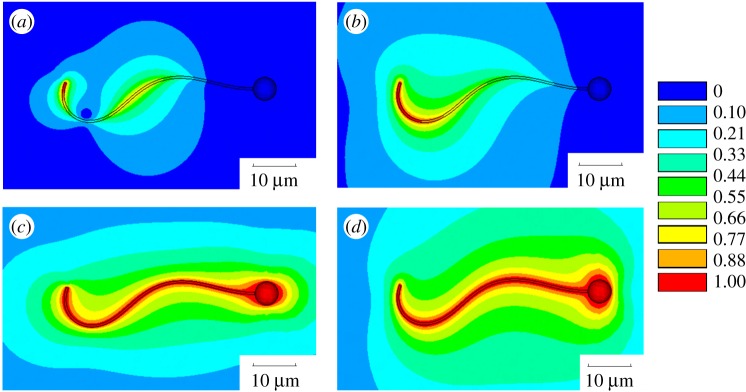
Absolute velocity distribution in the plane of the swimmer for four elementary motions: (*a*) a flexibly moving flagellum in the reference frame fixed with the swimmer head, (*b*) the swimmer rotating about the head centre as a rigid body, (*c*) the swimmer is rectilinearly moving in the streamwise direction as a rigid body and (*d*) the swimmer is rectilinearly moving in the transverse direction as a rigid body.

For each waveform configuration, a converged flow solution is obtained with applying non-slip condition on the swimmer surface with the velocity of the fluid equal to the velocity of the swimmer boundary, which consists of the rigid head and the flexible flagellum parts, and the full slip condition at all external boundaries. The solution obtained is found to be virtually insensitive to any further increase of the computational domain size or a further grid refinement. By integrating the forces on the flagellum surface, the drag force components and the torque specified on the right-hand side of equations (2.12)–(2.14) is calculated. In a similar way, the coefficients for the left-hand side of the same equations, which correspond to the two elementary rectilinear motions in the plane of the swimmer and the elementary rotation of the swimmer around its head centre as of a rigid body, are computed. These amount to four boundary value problems, which correspond to the same governing equations (2.24) and (2.25), the same computational domain, but different boundary conditions. These four problems are solved numerically with a FEM for each time moment in accordance with a particular phase of the swimming cycle (figure 4). Details of the FEM methods for numerical solution are summarized below. Following the standard approach [35], the FEM with second-order base functions is implemented in the framework of the penalty method, which requires minimization of the following functional:

$$\begin{array}{rl} J(u,v,w) & =\lambda \int_{V}{\left(\Delta \right)}^{2}\;dV+2\mu \int_{V}\left(\epsilon_{xx}^{2}+\epsilon_{yy}^{2}+\epsilon_{zz}^{2}+\frac{1}{2}\epsilon_{xy}^{2}+\frac{1}{2}\epsilon_{xz}^{2}+\frac{1}{2}\epsilon_{yz}^{2}\right)dV\\ & \;-\int_{V}(f_{x}u+f_{y}v+f_{z}w)\;dV \end{array}$$

with penalty parameter λ , where *ε*_*xx*_, *ε*_*yy*_, *ε*_*zz*_, *ε*_*xy*_, *ε*_*xz*_, *ε*_*yz*_ are components of the strain rate tensor

$$\begin{array}{rl} \left(\epsilon_{xx},\epsilon_{yy},\epsilon_{zz},\epsilon_{xy},\epsilon_{xz},\epsilon_{yz}\right) & =[\frac{\partial u}{\partial x},\frac{\partial v}{\partial y},\frac{\partial w}{\partial z},\frac{1}{2}\left(\frac{\partial u}{\partial y}+\frac{\partial v}{\partial x}\right),\frac{1}{2}\left(\frac{\partial u}{\partial z}+\frac{\partial w}{\partial x}\right),\frac{1}{2}\left(\frac{\partial v}{\partial z}+\frac{\partial w}{\partial y}\right)];\\ \Delta & =\frac{\partial u}{\partial x}+\frac{\partial v}{\partial y}+\frac{\partial w}{\partial z}; \end{array}$$

and *f*_*x*_, *f*_*y*_, *f*_*z*_ are internal forces. This results in a sparse system of linear algebraic equations that is solved using a direct method based on lower–upper (LU) decomposition. The magentablackIntel Math Kernel Library solver is used for solution of the linear system of equations.

## 3. Numerical results and data analysis

**Figure 5.**
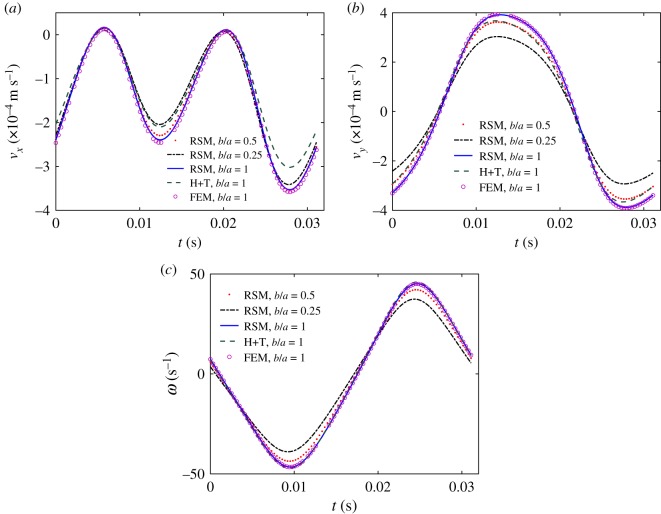
*v*_*x*_, *v*_*y*_ components of the velocity in the swimmer frame of reference (*a*,*b*) and angular velocity (*c*) for a cell with a spherical head (solid-blue, circle magenta and dashed-green) or an ellipsoidal head with aspect ratio 0.5 (dotted-red) and 0.25 (dashed-dot black). The circle-magenta points have been computed by an FEM code for comparison, the dashed-green curve were computed by using the simplified approach discussed in §3.2. The frame of reference for these calculations is located on the head centroid.

**Figure 6.**
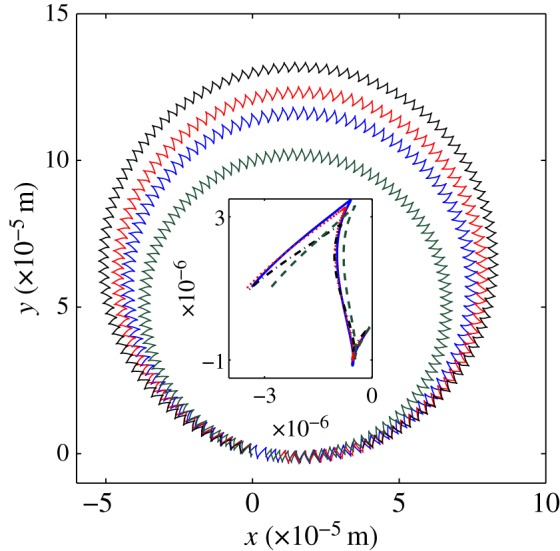
Swimming trajectory on the x–y plane for 107 beating periods and one period (inset) for a swimmer with a spherical head (solid blue) and a swimmer with an ellipsoidal head with aspect ratio equal to 0.5 (dotted red) and 0.25 (dashed-dotted black). In dashed-green, for comparison, the trajectory of a swimmer with a spherical head computed by approximating the resistive matrix coefficients as the sum of the head and flagellum separately. Note the smaller radius of the trajectory corresponding to larger coefficients $C_{x}^{v_{x}}$, $C_{y}^{v_{y}}$ (table 4). These are the trajectories for the head centroid, the axes units are in metres.

In figure 5, we plot the *x*- and *y*-components of the velocity and the *z*-component of the angular velocity in the frame of reference of the swimmer for different numerical methods and head shapes. Different points on the swimmer body draw different trajectories on the *x*–*y* plane; in figure 6 we display the trajectories of the head centroid.

An inspection of the frequency spectra of *v*_*x*_, *v*_*y*_ and *ω* reveals that the signal can be reconstructed within a 0.6% error by retaining the first five terms of the Fourier series expansion: $\sum_{k=0}^{4}a_{k}\cos(k\nu t+\phi_{k})$. The error is computed as: max|*v* − *v*_reconstructed_|/max|*v*|, where *v*_reconstructed_ is the reconstructed signal from the truncated Fourier series and *v* the original signal. When the parameter *K*_0_ is set to zero (the rectilinear swimmer) the curves *v*_*y*_ and *ω* have zero mean and are described within a 0.6% error by the first and third mode; keeping only the mode *k* = 1 guarantees an 8% error on *v*_*y*_ and a 6% error on *ω*. Differently, *v*_*x*_ is described within a 0.3% error by an expansion in the even modes *k* = 0, 2, 4. We have additionally verified for the rectilinear swimmer that the temporal variation of the angular frequency of the swimmer, *ω*, obtained numerically can be reasonably well approximated (within 6%) by a single harmonic function of the beating frequency, *ν*, regardless of the numerical discretization applied (e.g. 100, 200 and 400 points per the flagellum length).

We stress that since the system is linear no mechanism is in place to allow for the creation of non-zero modes from the forcing/boundary condition, in fact, the non-zero modes detected in *v*_*x*_, *v*_*y*_ and *ω* reflect the complex spectrum of $u_{x}^{BC}$ and $u_{y}^{BC}$ (equation (2.22)), which produces a coupling of the *x*- and *y*-coordinates of the local reference system of the flagellum. The fact that for the rectilinear swimmer only the even modes are excited in *v*_*x*_ and the odd ones in *v*_*y*_ matches the $u_{x}^{BC}$ and $u_{y}^{BC}$ spectra for the special case *K*_0_ = 0 as discussed at the end of §2.3. Similar considerations hold when comparing the spectra of $F_{x}^{B}$, $F_{y}^{B}$ $T_{z}^{B}$, that is the known term of the resistive matrix system and the unknowns *v*_*x*_, *v*_*y*_, *ω*.

As a preliminary validation, we compare the results obtained by means of the regularized Stokeslet method with those attained through the FEM [21]: see the difference in the resistive matrix coefficients (table 1), and compare the blue solid line and the magenta circles in figure 5. The difference between the coefficients is within a few per cent, while the differences between the velocities, quantified in table 2, are barely distinguishable and of second order when compared with the effects of the head shape or the errors introduced by the simplified approaches (dashed-green curve). This consistency guarantees the accuracy of our results and the reliability of both the numerical schemes.

**Table 1.** Difference between the resistive matrix coefficients and known terms of system (2.12)–(2.14) computed with the RSM and the FEM for a spherical head swimmer. The difference is computed as $\sum (C_{\mathrm{RSM}}-C_{\mathrm{FEM}})/\sum (C_{\mathrm{RSM}}+C_{\mathrm{FEM}})/2*100$, where the sum is performed over the time discretization and *C* refers to any of the coefficients considered. The second row in this table indicates the order of magnitude of the corresponding coefficient.

$C_{x}^{v_{x}}$	$C_{x}^{v_{y}}$	$C_{x}^{\omega }$	$C_{y}^{v_{x}}$	$C_{y}^{v_{y}}$	$C_{y}^{\omega }$	$m_{z}^{v_{x}}$	$m_{z}^{v_{y}}$	$m_{z}^{\omega }$	$F_{x}^{B}$	$F_{y}^{B}$	$T_{z}^{B}$
∼ 1 × 10^−4^	∼ 1 × 10^−6^	∼ 1 × 10^−10^	∼ 1 × 10^−6^	∼ 1 × 10^−4^	∼ 1 × 10^−9^	∼ 1 × 10^−10^	∼ 1 × 10^−9^	∼ 1 × 10^−14^	∼ 1 × 10^−8^	∼ 1 × 10^−8^	∼ 1 × 10^−12^
2.92%	−5.5%	1.55%	−5.5%	1.49%	0.9%	1.55%	0.9%	3.69%	−1.76%	−3.58%	1.06%

**Table 2.** Values of the time averaged and rescaled root mean square error for the swimming velocities computed with the RFT-L, the RFT-GH, the head + tail (H + T) and the FEM model. The results are for the rectilinear (*K*_0_ = 0 rad s^−1^) and curved (*K*_0_ = 7735.5 rad s^−1^) swimmer with λ = λ_0_.

	rad s^−1^	*v*_*x*_ RMSE	*v*_*y*_ RMSE	*ω* RMSE
RFT-GH,	*K*_0_ = 0	0.1577	0.1103	0.06478
RFT-L,	*K*_0_ = 0	0.08814	0.06211	0.04796
RFT-GH,	*K*_0_ = 7735.5	0.1337	0.1102	0.06492
RFT-L,	*K*_0_ = 7735.5	0.07523	0.06196	0.04787
H + T,	*K*_0_ = 7735.5	0.06963	0.03545	0.000632
FEM,	*K*_0_ = 7735.5	0.01448	0.00334	0.001277

Compared with the RSM, the FEM calculation is much more expensive since it solves the governing equations discretized in the entire flow domain and not just on the swimmer’s surface. For example, the RSM calculations performed here took several minutes per case on a single processor. For the FEM calculation, the same required about 35 h with running two OpenMP threads in parallel. The amount of computer memory in each case was more comparable: 15 Gb for the RSM method and 27 Gb for the FEM solution per case.

We have verified that for the calculations presented in this paper the motility matrix coefficients are symmetric within numerical precision as dictated by the reciprocal theorem.

As a further remark, note that the curvature parameter *K*_0_ > 0 of the swimmer waveform in (2.20) corresponds to a circular trajectory in the absolute frame of reference as shown in figure 6. Accordingly, this should give rise to apparent accelerations in the swimmer’s frame, which are not accounted for in the Stokes model. To justify the neglect of these accelerations, we want to evaluate the order of magnitude of these terms first. The difference between the accelerations in the non-inertial frame **a**_*r*_ and those in the inertial frame **a**_*f*_ are

3.1 $$a_{r}-a_{\;f}=-\omega \times (\omega \times r)-2\omega \times v-\dot{\omega }\times r,$$

where the first term on the right-hand side represents the centrifugal acceleration, the second the Coriolis acceleration and the third the Euler acceleration. The force associated to **a**_*r*_ in our calculations is at most of the order $O(1\times 10^{-15})$, that is three orders of magnitude smaller than the smallest coefficients in the motility matrix, thus negligible as initially hypothesized. Still, it can be argued that even a small unbalanced force can build up into a non-negligible effect for the swimmer trajectory over a time period long enough compared with the swimmer cycle. Therefore, to confirm that the effect of the non-inertial forces on the trajectory of the swimmer is small, we compared the swimmer’s trajectories with and without taking the apparent accelerations into account in accordance with the ‘instantaneous’ coordinate and velocity of the swimmer calculated numerically. Over a few circular trajectory periods, the swimmer trajectories with and without taking the apparent accelerations into account virtually coincided, which finally justifies the neglect of these terms.

### 3.1. Resistive force theory

According to the RFT, the viscous forces applied to the flagellum centreline depend on the flagellum velocities through

3.2 $$f=[K_{N}I-(K_{T}-K_{N})tt^{T}]\cdot u^{BC},$$

where *K*_T_ and *K*_N_ are the tangential and normal friction coefficients, **t** = (cos*Ψ*, sin*Ψ*) the tangent to the flagellum centreline and I the identity matrix. Equation (3.2) can alternatively be expressed as [21]

3.3 $$f=RKR^{-1}u^{BC}$$

where R is the rotation matrix

3.4 $$\left[\begin{array}{cc} \cos\Psi & -\sin\Psi \\ \sin\Psi & \cos\Psi \end{array}\right]$$

and K is a diagonal matrix with *K*_T_ and *K*_N_ on the diagonal. We proceed by (i) substituting the expressions for *Ψ* and **u**^*BC*^ provided in §2.3 in (3.2) or (3.3), (ii) computing the coefficients of the propulsion matrix and (iii) solving the linear system (2.12)–(2.14). In point (ii), the friction coefficients are computed as the sum of the head and tail contribution as expressed by (2.15). The head contribution is known analytically for a spherical or ellipsoidal head, while the tail friction coefficients are computed by integrating numerically (3.2) or (3.3) and the *z* component of the torque **x** × **f** for the entire flagellum length after replacing **u**^*BC*^ by, in turn, a unitary forward and transversal velocity and an unitary angular velocity.

Following [18] we contrast the results for two possible choices of the friction coefficients: those derived by Lighthill (RFT-L) [16] and those suggested by Gray & Hancock (RFT-GH) [17]

3.5 $$K_{T,L}=\frac{2\pi \mu }{\ln(0.18\lambda /F_{r})},\;K_{N,L}=\frac{4\pi \mu }{\ln(0.18\lambda /F_{r})+1/2}$$

and

3.6 $$K_{T,\mathrm{GH}}=\frac{2\pi \mu }{\ln(2\lambda /F_{r})-1/2},\;K_{N,\mathrm{GH}}=\frac{4\pi \mu }{\ln(2\lambda /F_{r})+1/2}.$$

**Figure 7.**
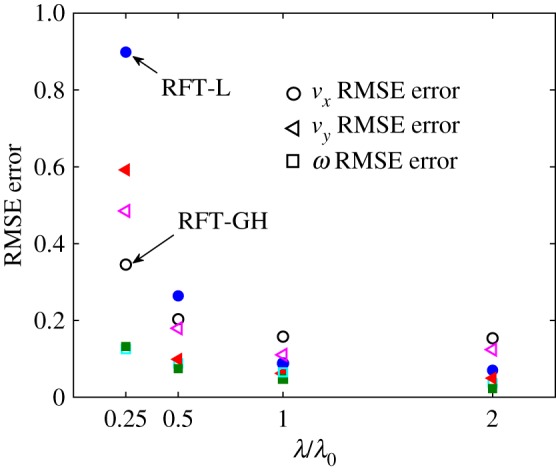
RMSE averaged over one period for the swimming velocities (*v*_*x*_-circles, *v*_*y*_-triangles) and the angular velocity (*ω*-squares) for the RFT Lighthill (RFT-L, filled symbols) and the RFT Gray and Hancock (RFT-GH, empty symbols) model. The error is plotted as a function of the wavenumber λ normalized by the reference wavenumber λ_0_ (see formula (2.20)). The results are for the rectilinear swimmer. In table 2, we report the numerical value of the errors for λ = λ_0_ and some extra cases.

We also compare the results obtained with the RFT with those obtained with the RSM. In figure 7 and table 2, we quantify the error of the former as

$$\text{RMSE}=\frac{\sqrt{\left\langle {\left(u_{\mathrm{RFT}}-u_{\mathrm{Stk}}\right)}^{2}\right\rangle }}{\left(\max u_{\mathrm{Stk}}-\min u_{\mathrm{Stk}}\right)},$$

where ‘⟨ ⟩’ is the average over one beating period, *u*_Stk_ refers to the value computed with the regularized Stokeslet model, and the denominator rescales the root mean square error by the range of variability of the quantity under consideration, being it *v*_*x*_, *v*_*y*_ or *ω*. For the reference case of the swimmer with λ = λ_0_ (solid blue line in figure 5), we find that Lighthill coefficients outperform Gray and Hancock’s both for the curved and rectilinear swimmer (figure 7 and table 2). However, the model performances depend on the geometry, as clearly seen in figure 7. For λ < λ_0_, we find that the RFT-GH model gives better answers than RFT-L and for λ = λ_0_/4 both RFT models are deemed unreliable. We have also checked that the simplified model referred to as the head + tail model (H + T), which will be discussed in the next section, performs better than the RFT (table 2).

Note that, as stressed in [20], what really matters to reproduce the kinematics of the motion correctly is the ratio between the tangential and the normal friction coefficients, *K*_N_/*K*_T_. The choice of the friction coefficients for the head is also only important in relative terms: the absolute values of $C_{x}^{v_{x}}$ and $C_{y}^{v_{y}}$ are irrelevant as far as they maintain the right proportion with one another and the flagellum coefficients, this is because the kinematics results from a force balance. The choice of using Perrin’s formulae for the Brownian motion of an ellipsoid provides coefficients whose ratio is comparable to the ratio for an ellipsoid in a Stokes flow as given by (2.16) and (2.17) and whose absolute values (at least for the *b*/*a* = 0.5 aspect ratio) are not very dissimilar (table 3). However, given the size and speeds involved, resorting to the Stokes law seems more physically based. Table 3 reveals that only the RFT-L model has both *K*_T_ and *K*_N_/*K*_T_ within the range indicated in [20]. While in [20] it is found that the RFT reproduces the trajectories reliably, in [18] it is reported that the drag values computed with RFT are inaccurate. This may not necessarily be in contrast since for the last calculations it is the absolute value of the coefficients that matters. However, it is most likely the case that while the customarily chosen RFT coefficients well fit the data for the swimming of a spermatozoon with reference parameters, they do not match the results for different geometries, e.g. larger or smaller λs or helical flagella as in [18].

**Table 3.** Values of the longitudinal friction coefficients for a unitary viscosity (the values from [12,20] are divided by 0.7 mPa s) and ratio between the normal and longitudinal friction coefficients for the head and flagellum. Following [12,20] the head friction coefficient is computed for an aspect ratio *b*/*a* = 0.5 and *L*_head_ = 10 *µ*m.

	$C_{x}^{v_{x},h}$	$C_{y}^{v_{y},h}$/$C_{x}^{v_{x},h}$	*K*_T_	*K*_N_/*K*_T_
	N s m^−1^	—	N s m^−2^	—
Stokes and RFT-L	5.6734 × 10^−5^	1.14532	1.7326	1.76
Stokes and RFT-GH	5.6734 × 10^−5^	1.14532	1.1353	1.69
Perrin’s formula and *K*_N_ and *K*_T_ as in [20]	5.7571 × 10^−5^	1.14392	0.9857 ± 0.8857	1.81 ± 0.07
Perrin’s formula and *K*_N_ and *K*_T_ as in [12]	5.7571 × 10^−5^	1.14392	0.5429	1.89

### 3.2. Inaccuracy of treating the head and flagellum separately and further comparisons with the RFT solutions

Although being accurate compared with the RFT, our results indicate that the simplified model, consisting in evaluating the friction coefficients as the sum of the head and the tail contribution calculated independently, leads to notable errors. This approximation is convenient for first estimates since it reduces the computational cost when analytical solutions are available, e.g. for spherical or ellipsoidal heads, but neglects the interaction between the tail and the head. The system (2.8), in short, $u^{BC}+v_{\;j}+\omega \times x\cdot e_{\;j}=u^{\mathrm{tot}}=Jf$, is inverted into $f=J^{-1}u^{\mathrm{tot}}$, in this form each component of the vector of local forces **f**, being it located on the head or on the tail, can be split up into two contributions: one due to the points located on the head and one due to the points located on the tail. With superscript ‘*h*’ and ‘*t*’ denoting the head and tail, respectively, and greek letters indicating the points on the surface of the swimmer we have

$$f_{\alpha^{h}}=J_{\alpha^{h}\beta^{h}}^{-1}u_{\beta^{h}}^{\mathrm{tot}}+J_{\alpha^{h}\beta^{t}}^{-1}u_{\beta^{t}}^{\mathrm{tot}}$$

and

$$f_{\alpha }^{t}=J_{\alpha^{t}\beta^{h}}^{-1}u_{\beta^{h}}^{\mathrm{tot}}+J_{\alpha^{t}\beta^{t}}^{-1}u_{\beta^{t}}^{\mathrm{tot}},$$

the approximate approach neglects the head–tail interaction terms: $J_{\alpha^{h}\beta^{t}}^{-1}u_{\beta^{t}}^{\mathrm{tot}}$ and $J_{\alpha^{t}\beta^{h}}^{-1}u_{\beta^{h}}^{\mathrm{tot}}$.

For the case of a spherical head, we have verified that the approximate method overestimates the leading coefficients of equations (2.12) and (2.13), $C_{x}^{v_{x}}$ and $C_{y}^{v_{y}}$, by approximately 23.5% and 17% and incorrectly captures many others (table 4). The difference between the resistive matrix coefficients calculated with the two methods is expressed by

3.7 $$\frac{\int_{0}^{T}(C_{\mathrm{head}-\mathrm{off}}-C_{\mathrm{head}-\mathrm{on}})\;dt}{\int_{0}^{T}C_{\mathrm{head}-\mathrm{on}}\;dt}*100,$$

where *C* refers to any coefficient and known term of equation (2.12)–(2.14). A positive value signifies that on average the simplified approach overestimates the coefficient. Remarkably, the overestimate of $C_{x}^{v_{x}}$ and $C_{y}^{v_{y}}$ leads to swimming trajectories with different radii as shown in figure 6 (compare the solid blue and dashed-green curve).

**Table 4.** Difference between the resistive matrix coefficients and known terms of system (2.12)–(2.14) computed with the simplified assumption of representing the swimmer as the superposition of the head and tail flow field (resistive matrix (2.15)) and the full-swimmer representation. The error is computed according to formula (3.7). The second row in this table indicates the order of magnitude of the corresponding coefficient.

$C_{x}^{v_{x}}$	$C_{x}^{v_{y}}$	$C_{x}^{\omega }$	$C_{y}^{v_{x}}$	$C_{y}^{v_{y}}$	$C_{y}^{\omega }$	$m_{z}^{v_{x}}$	$m_{z}^{v_{y}}$	$m_{z}^{\omega }$	$F_{x}^{B}$	$F_{y}^{B}$	$T_{z}^{B}$
∼ 1 × 10^−4^	∼ 1 × 10^−6^	∼ 1 × 10^−10^	∼ 1 × 10^−6^	∼ 1 × 10^−4^	∼ 1 × 10^−9^	∼ 1 × 10^−10^	∼ 1 × 10^−9^	∼ 1 × 10^−14^	∼ 1 × 10^−8^	∼ 1 × 10^−8^	∼ 1 × 10^−12^
23.47%	−4.25%	4.53%	−4.25%	17.21%	5.7%	4.53%	5.7%	−0.59%	7.07%	2.9%	−0.26%

**Figure 8.**
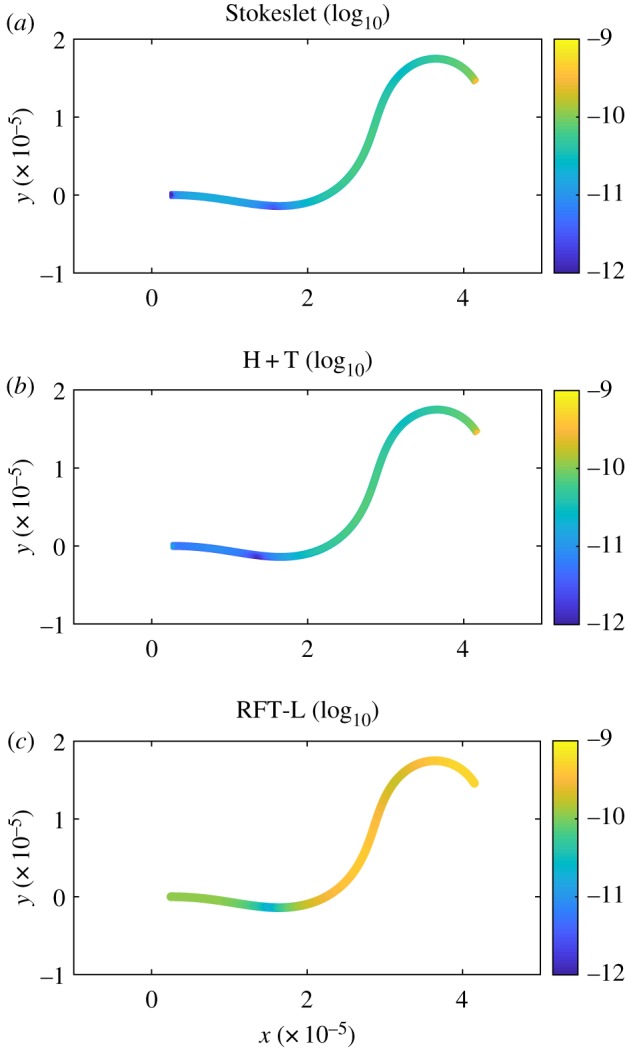
Logarithm with base 10 of the modulus of the forces on the surface of the flagellum for the full-body regularized Stokeslet model (*a*), the head + tail model (*b*) and the RFT with Lighthill’s coefficients (*c*). For the first two models the forces are distributed on the cylindrical surface of the flagellum whose radius is *F*_*r*_ = 0.25 μm, while for the RFT forces are distributed on the flagellum centreline. Figures are in logarithmic scale to highlight the force distribution on the entire extension of the tail; note, in fact, that the values at the tip of the tail are the largest and a linear scale would only stress this result.

The simplified approach results in a different distribution of the forces on the surface of the flagellum as shown in figure 8; note that the values differ in particular on the left end where the flagellum is attached to the head. In the full-body model the values are zero because of the presence of the boundary, while in the simplified model the values are relatively large since this is a free end; however, they are not as high as on the tip of the tail given the lower velocities. Note again that even if inaccurate, the head + tail model clearly outperforms the RFT (figure 8*c*), for which the force distribution only qualitatively resembles the first two models.

**Figure 9.**
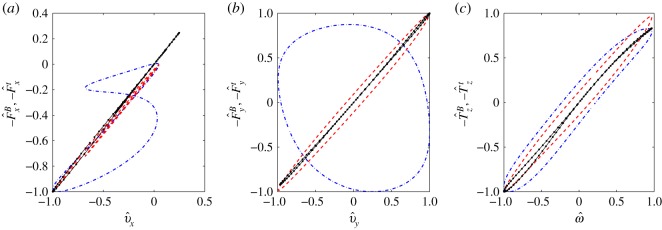
In dashed-red: surface integral on the swimmer flagellum of minus the *x*- and *y*-components of the force $-F_{x}^{t}$, $-F_{y}^{t}$ (*a*,*b*) and *z*-component of the torque $-T_{z}^{t}$ (*c*), versus, respectively, the swimmer velocity in *x*, *y*, and the angular velocity. In dotted-dashed blue and dotted black, we show similar curves for the integral of the forces on the flagellum due to the beating motion only (right-hand side of system (2.12)–(2.14)), the dotted black case corresponds to the values in the coordinates of the diagonal system. All the variables in these plots are normalized by their maximum absolute value and for this reason are marked by a hat.

In figure 9, we plot in dashed-red the total forces and torque exerted by the head to the tail versus the swimmer linear and angular velocities. These results were obtained for the full-body regularized Stokeslet model calculation. We find that the points do not lie on a straight line as would be expected for the flow past an isolated sphere or ellipsoid, instead, they trace closed curves. The deviation from a straight line quantifies the contribution due to the head–tail interaction and further demonstrates the limitations of the approximate approach. Lines fitted to the dashed-red curves of figure 9 have slopes smaller than the theoretical coefficient 6*πμa* by 8.5% in *x*-direction, larger by 9.8% in *y*-direction and smaller than 8*πμa*^3^ by 6.9% for the torque in *z*. Similar results hold when comparing the theoretical friction coefficients for prolate ellipsoids with the slopes of lines fitted to analogous curves for swimmers with ellipsoidal heads.

### 3.3. Sensitivity to the head shape

We study how swimming is affected by the head shape; in particular, we consider the case of swimmers with a prolate spheroidal head. We keep the head volume constant while varying the minor to major axis ratio *b*/*a*. In figures 5 and 6, we report results for ellipsoidal heads with an aspect ratio of 0.5 and 0.25 (dotted red and dashed-dotted black curves). Despite the identical beating movement, the tail integral of forces and torque varies as the head shape varies, given the different swimming velocities and head–tail interaction.

**Figure 10.**
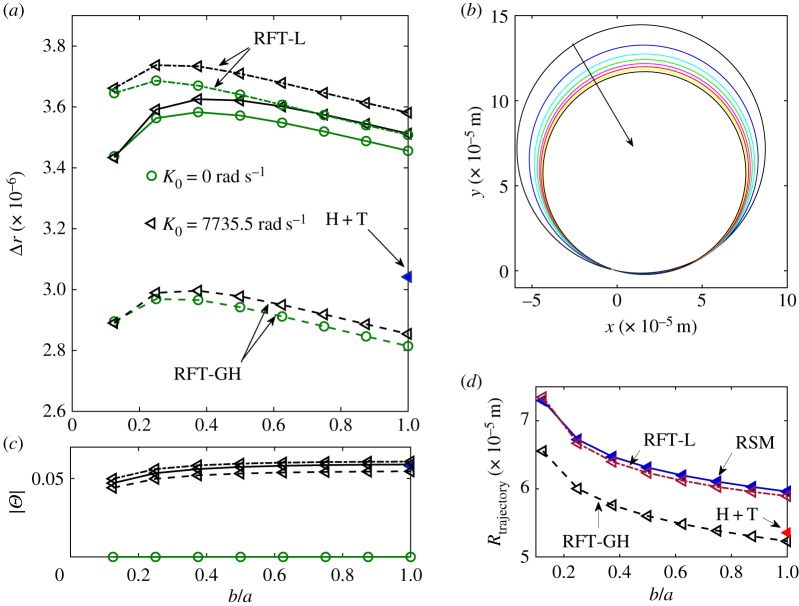
Net displacement (*a*) and rotation angle (*c*) for one beating period as a function of the aspect ratio that characterizes the ellipsoidal head. The curves correspond to the case of null (green circles) and non-null (black triangles) mean flagellar curvature and the RSM (solid line), the RFT Lighthill model (dashed-dotted line), the RFT Gray and Hancock model (dashed-line) and the head + tail model (filled triangle). (*b*) Swimming trajectories on the *x*–*y* plane, drawn by using the values of the net displacement, Δ*r*, and net rotation angle, *Θ*, for the swimmer with non-null mean curvature. The arrow indicates the direction of growing *b*/*a*. (*d*) Radius of the trajectory as a function of *b*/*a* for the regularized Stokeslet model (solid line), the RFT Lighthill model (dash-dotted line) and the RFT Gray and Hancock model (dashed line), the red triangle refers to the head + tail model.

We have already quantified in figure 7 and table 2 the error of the RFT in predicting the absolute value of the velocity, here we report, in addition, a slight discrepancy between the RFT and the regularized Stokeslet model in identifying the fastest swimmer. In figure 10, we plot the net displacement and rotation angle *Θ* for one period as a function of the minor to major axis ratio *b*/*a*. Note that for both values of *K*_0_ the swimmer that swims the farthest is the one with *b*/*a* = [0.375 0.5] for the Stokeslet model, *b*/*a* = 0.25 for the RFT models. We have estimated the error bar associated with the chosen numerical resolution of RSM to be 0.42%. This value corresponds to the *x*-velocity component error of the RSM solution for solving the analytical problem reported in figure 1 at the same numerical resolution as the swimmer problem. The *x*-velocity has been selected as the most sensitive solution component since it corresponds to the maximum discrepancy between the RSM solution and the reference FEM solution (table 2). The swimmers that possess the largest *v*_*x*_ average velocity according to the RSM are those with *b*/*a* = [0.625 1], while for the RFT models the maximum *v*_*x*_ average velocity is achieved for *b*/*a* = 0.625.

The results in figure 10*a*,*c* reiterate that for the wavenumber of choice the RFT-L model is more accurate than the RFT-GH model. Note that both the RFT models badly capture the behaviour for the most stretched shapes. Observe also that the swimmer with a non-null curvature swims farther than the rectilinear swimmer (covers larger distances in one period).

For the *K*_0_ = 3375.5 rad s^−1^ swimmer, the net displacement and angle *Θ* determine the curved trajectories reported on figure 10*b*. For a given displacement, larger angles correspond to trajectories with smaller radii, so that swimmers with more elongated heads display trajectories with larger radii. The behaviour is monotonic with *b*/*a* across all models (figure 10*d*), surprisingly, the RFT-L model predicts the trajectory radius even better than the head + tail model despite the lower accuracy in the velocities.

### 3.4. Flow field around the swimmer

**Figure 11.**
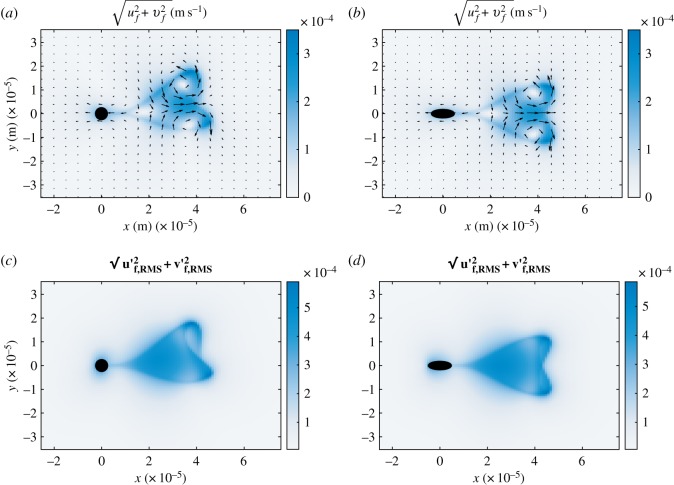
(*a*, *b*) Modulus of the average flow field (components *v*_*f*_, *u*_*f*_, *w*_*f*_ = 0) computed over a beating period in the frame of reference of the swimmer. The black arrows indicate the direction and magnitude of the local field. Panel (*a*) represents the case of the swimmer with non-zero curvature *K*_0_ = 7735.5 rad s^−1^, (*b*) the calculation for *K*_0_ = 0 and *b*/*a* = 0.375, that is, the fastest swimmer. In black, we mark the area occupied by the solid head. (*c*, *d*) Modulus of the root mean square value of the velocity fluctuations averaged over one period (equation (3.8)) for the same two cases of the top line.

In figure 11, we show the average flow field *u*_*f*_,*v*_*f*_ and the root mean square velocity fluctuations

3.8 $$\begin{array}{cc} u_{\;f,\mathrm{RMS}}^{\prime } & =\sqrt{\left\langle {\left(u_{\;f}-\langle u_{\;f}\rangle \right)}^{2}\right\rangle }\\ \text{and}\;v_{\;f,\mathrm{RMS}}^{\prime } & =\sqrt{\left\langle {\left(v_{\;f}-\langle v_{\;f}\rangle \right)}^{2}\right\rangle }, \end{array}\}$$

around the swimmer in the frame of reference of the swimmer, here the brackets denote the time average.

We compare the case of swimmers that draw circular and straight trajectories, and for the latter we report results for the swimmer with *b*/*a* = 0.375. The swimmer flow field resembles the pattern produced by two counterrotating vortex dipoles, one centred at the head, the other centred at about *x* = 4 × 10^−6^. Note that in figure 11*a*, which corresponds to the case of *K*_0_ = 7735.5 rad s^−1^, the second dipole structure is offset with respect to the first one, this reflects the asymmetric beating movement visualized in figure 2 and responsible for the curved trajectory.

**Figure 12.**
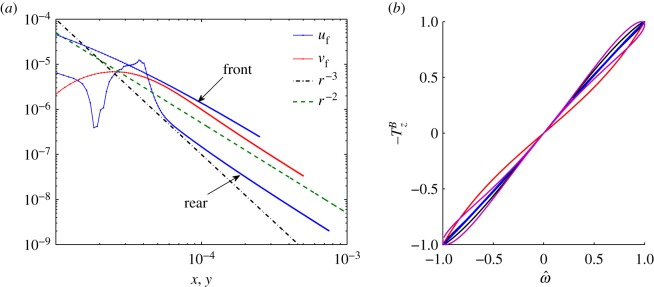
(*a*) Log–log plot of the *u*_*f*_ and *v*_*f*_ average velocity profile respectively along the *y* = 0 and *x* = 0 cross section on the *z* = 0 plane for the *K*_0_ = 0 swimmer with a spherical head. The *u*_*f*_ profile differs on the front and rear of the swimmer as marked by the arrows, while the *v*_*f*_ profile is symmetric with respect to the *y* = 0 line. Note the length over which the profile has been computed, one order of magnitude larger than the plots in figure 11, and the *r*^−2^ far field scaling. (*b*) Torque exerted by the flagellum due to the beating motion only versus the swimmer angular velocity for the diagonalized system and the cases: λ = λ_0_ (black), λ = λ_0_/2 (blue), λ = 2λ_0_ (magenta), λ = 100 m (red). All the variables in these plots are normalized by their maximum absolute value.

In figure 12, we display the average flow field profile for a cross section in *x* and *y* that spans the interval [0, 5 × 10^−4^] m, we observe the *r*^−2^ scaling emerging at large enough distances for respectively the *u*_*f*_ and *v*_*f*_ component of the velocity. Close to the swimmer the velocity profile does not follow a clear power law and the *u*_*f*_ component dominates.

### 3.5. Eigenvalues of the propulsive matrix system

The system of equations (2.12)–(2.14) can be diagonalized to remove the effect of the coordinate coupling. When doing so, the curves that on the $v_{\;j}-F_{\;j}^{B}$, $\omega_{\;j}-T_{\;j}^{B}$ planes draw close loops collapse almost perfectly, and somewhat surprisingly, to a single line as expected for bodies of fixed shape (e.g. a sphere whose line slope on the *v*_*j*_ − *F*_*j*_ plane would be 6*πμa*). See figure 9 and compare the dashed-dotted blue curve versus the black dots curve. The eigenvalues *Λ*^*x*^(*t*), *Λ*^*y*^(*t*), *Λ*^*z*^(*t*) and eigenvectors of the system are in general a function of time; however, the dotted-black curves of figure 9 can be well fitted by lines $-F_{x}^{B}\approx \Lambda^{x}v_{x}$, $-F_{y}^{B}\approx \Lambda^{y}v_{y}$, $-T_{z}^{B}\approx \Lambda^{z}\omega$ of slope *Λ*_*x*_ = 0.00011 N s m^−1^, *Λ*_*y*_ = 0.00013 N s m^−1^, *Λ*_*z*_ = 3.7 × 10^−14^ N s m. These are not the slopes that appear in figure 9, where quantities are normalized by their maximum absolute values to allow for comparison. As for the eigenvalues, only the *x* and *y* axes appear to change their orientation in time while the *z* axis along which *ω* and $T_{z}^{B}$ are directed is fixed.

In conclusion, despite the fact that the swimmer body goes through cyclical deformations, we are able to identify single time-independent friction coefficients *Λ*^*x*^, *Λ*^*y*^, *Λ*^*z*^ that in opportunely rotated frames (the diagonal ones) relate $F_{\;j}^{B}$ to *v*_*j*_, and *ω* to $T_{z}^{B}$. This fact is consistent with the assumption of a quasi-steady flow described by the time-independent Stokes equations.

However, for larger λ: λ = 2λ_0_ and λ = 100 m, the latter representing the case λ → ∞, the friction coefficients of the propulsion matrix show a more marked dependence on time since the points corresponding to the diagonalized system do not lie on a line but draw loops or S-shaped curves in the $\omega_{\;j}-T_{\;j}^{B}$ plane (figure 12*b*). Therefore, the flow is sensitive to the change of shape of the beating flagellum and this suggests that the quasi-steady and inertia-less assumptions may break down. It is important to point out that while the frequency Reynolds number is unchanged, the Reynolds number increases as λ increases given the larger swimming velocities. The highest Reynolds number for the λ = 100 m case is *Re* = 0.035.

## 4. Conclusion

We study numerically the motion of a flagellated microswimmer, specifically a sperm-cell swimmer, in an infinite domain. We first simulate locomotion by using two different numerical methods: the RSM and the FEM, finding a very good agreement between the two.

We find that the RFT performs reasonably well for swimming parameters close to laboratory observations: the normalized root mean square error for the swimming velocities is within 5–15% depending on the choice of the model parameters. However, the model is unreliable for smaller values of the wavelength, specifically, the case of wavelengths of about $\frac{1}{4}$ and $\frac{1}{2}$ of the total flagellum length, while the reference case has a wavelength of about one flagellum length. These results are consistent with the findings of previous studies that were focused on other types of flagella such as prokaryotic or bacteria-like type [18,19,36]. For example, in [18] it was reported that RFT fails to provide an accurate description of helical shapes relevant to bacteria, while in [36] it was concluded that for the broad range of geometry parameters of helical flagellated swimmers considered, RFT never gives accurate results. It was also pointed out that despite being unable to capture the full dynamics, RFT sometimes provides accurate solutions for single quantities [36]. This is an observation that we also made in reference to the RFT model with the choice of parameters suggested by Lighthill which is able to accurately predict the swimming trajectory radius. Finally, in [19] it was noted that when studying the complex geometry of superhelices, the experimental results are in excellent agreement with the calculations performed with the RSM, which is not the case of the RFT model. We also find that the RFT approach fails to correctly predict the optimum shape of the swimmer’s head for fastest swimming in the rectilinear case.

We find that the simplified approach that consists in studying swimming as the linear superposition of the head and the tail contribution separately, referred to as the head + tail model, leads to inaccurate results and we precisely quantify the error for the velocities, trajectory and force distribution. The inaccuracy of the method originates from the fact that the interaction terms between the head and the flagellum are neglected, or, else, from a physical perspective, the model fails to account for the front–rear symmetry breaking of the flow past the ellipsoidal head due to the presence of the flagellum. For a circular swimmer the radius difference between the trajectory of the head + tail and full-body model is comparable to the radius difference between the trajectory of a swimmer with a spherical head and a swimmer with an ellipsoidal head of minor to major axis ratio 0.25. This suggests that this simplified approach as well as the RFT is unsuitable to perform optimization studies on hydrodynamically efficient body and beating shapes.

Finally, we have revealed by diagonalizing the propulsion matrix that the flow is rather insensitive to the cyclic deformation of the swimmer body for our choice of parameters *K*_0_, *A*_0_ and λ_0_. In fact, during one swimming cycle the points that correspond to different instant of time and therefore different shape configurations, lie on an almost perfect straight line on the *v*_*x*_-$F_{x}^{B}$, *v*_*y*_-$F_{y}^{B}$ and *ω*_*z*_-$T_{z}^{B}$ plane, a behaviour typical of fixed-shape objects for which the friction coefficients are given. Consider, however, that the friction coefficient is approximately constant provided that the axes are opportunely rotated as the flagellum moves, this is where the analogy with fixed-shape objects ends. We have also observed that if we fix the frequency Reynolds number and change the wavenumber λ of the flagellum travelling wave by choosing larger values, the curves on the *ω*_*z*_-*T*_*z*_ plane start displaying a markedly nonlinear behaviour. This suggests that for these cases the flow ‘sees’ the object deforming and hence could be prone to time- and inertia-dependent behaviours. This also calls for a more accurate definition of the relevant Reynolds number in the case of the flagellated swimmers compared with those two commonly used in the literature, which are based on the swimmer length or its beating frequency.

## Supplementary Material

Data relative to Figures 5, 6, 8, 9, 10, 11, 12

## Supplementary Material

Flow Field generated by the beating flagellum

## Supplementary Material

Flow Field generated by a unitary rigid rotation of the swimmer body

## Supplementary Material

Flow Field generated by a unitary rigid translation of the swimmer body along x

## Data accessibility

Additional data is provided as Electronic Supplementary Material.
